# Effect of Cellulose Nanofibrils on the Physical Properties and Frost Resistance of Pervious Concrete

**DOI:** 10.3390/ma15227906

**Published:** 2022-11-09

**Authors:** Xu Zhang, Chengbang Lei, Zhi Li, Aiqin Zhang, Wanfeng Zhao, Wei Zhang, Jiarong Xu, Panpan Guo

**Affiliations:** 1 School of Transportation Civil Engineering, Shandong Jiaotong University, Jinan 250357, China; 2 School of Civil Engineering, Shandong Jianzhu University, Jinan 250101, China; 3 Engineering Research Institute of Appraisal and Strengthening of Shandong Jianzhu University Co., Ltd., Jinan 250014, China

**Keywords:** pervious concrete, cellulose nanofibrils (CNFs), rheology, strength, permeability, frost resistance

## Abstract

Pervious concrete has good water permeability and, if used in construction, it can alleviate the heat island effect. However, its low strength and poor durability are major obstacles to its use. This study shows that nano-reinforced pervious concrete created by incorporating cellulose nanofibrils (CNFs) can improve the physical properties and increase the durability of pervious concrete. CNFs were added to the concrete mix in proportions ranging from 0.05% to 0.2% by weight of binder. The additions were found to alter matrix rheology. The hydration kinetics of matrix with differing CNF contents were compared and analyzed. The experimental results show the addition of CNFs delayed peak heat flow and maximum cumulative heat. The 28 d compressive strength of pervious concrete increased by up to 26.5% and 28 d flexural strength by up to 25.8% with the addition of 0.05–0.2% CNFs. Addition of 0.1% and 0.2% CNFs increased water permeability. Addition of 0.05–0.15% CNFs decreased mass loss by 73.2–83.7% after 150 freeze–thaw cycles, which corresponded to an increase in frost resistance. Denser matrices and stronger interfacial transition zones were observed using scanning electron microscopy when 0.05–0.2% CNFs were added.

## 1. Introduction

Pervious concrete alleviates the urban heat island effect, thereby improving the urban environment and reducing the severity of environmental problems caused by concrete. Pervious concrete utilized in urban pavements and highways can allow stormwater to pass easily from the surface to subsurface through the interconnected pores, thus avoiding waterlogging. The collected stormwater is further used for the natural recharge of soil. Meanwhile, large interconnected pores can promote the evaporation of water from underlayers. Nevertheless, ordinary pervious concrete usually has poor mechanical properties and low frost resistance due to its high porosity, which can present problems when the concrete is in an extremely wet or cold environment. Researchers have directed attention to producing pervious concrete with high strength, high durability, and excellent water permeability.

Traditional methods to improve the strength and durability of pervious concrete include optimizing aggregate size distribution and modifying the aggregate–paste interfacial transition zone (ITZ). A smaller aggregate size or a lower proportion of aggregates increases the strength of pervious concrete but also significantly decreases permeability [1]. Agar-Ozbek et al. [2] investigated the influence of different aggregate particle sizes on specimen cracks using microtomography. When aggregate particle size was small, the cracks mainly appeared in the paste, but when the aggregate was large, cracks appeared mainly at the ITZ. The crucial unresolved task is to improve the mechanical properties and durability of pervious concrete without significantly decreasing permeability. The incorporation of additives or composites such as ground granulated blast furnace flag (GGBS), silica fume (SF) or fly ash (FA) can increase matrix compactness and strengthen the ITZ, thus increasing the mechanical strength and durability of pervious concrete without a significant reduction in permeability. Li et al. [3] made high-strength permeable concrete pavement with a compressive strength of 61 MPa and a permeability coefficient of 13.02 mm/s using mineral powder (SLAG) and fly ash (FA). Tabatabaeian et al. [4] found that an optimal mix of epoxy resin and different types of coarse aggregate increased mechanical properties as well as freeze–thaw resistance and permeability.

Recent research into the application of nanomaterials has produced promising techniques that improve the mechanical properties and durability of concrete. Nanocellulose, an environmentally friendly and renewable nanomaterial, has been increasingly used in cement-based materials. Nanocellulose originates from plant or tree wastes, providing an environmental alternative admixture for cement-based materials. The main forms of nanocellulose are cellulose nanocrystals (CNCs) and cellulose nanofibrils (CNFs), divided by aspect ratio, crystallinity and so on. They have the advantages of high aspect ratio, high elastic modulus, low density, high specific surface area and good thermomechanical stability, and show great potential in this field. Due to the ability of nanocellulose to absorb water, the rheological properties of fresh cement matrix can be adjusted [5]. Thus, nanocellulose as viscosity-modifying agents have been used to improve the mixture stability of self-consolidating concrete [6] and the fiber dispersion for engineered cementitious composites [7,8]. The absorption of nanocellulose on the surface of cement particles may affect the hydration kinetics of cement. Cao et al. [9] investigated the effect of CNCs on cement hydration, and they found that CNCs increased cumulative heat release but delayed the arrival of peak heat release. They hypothesized that short-circuit diffusion in CNCs explained the hydration effect of CNCs. Fu et al. compared the effect of CNCs from different raw material sources on the hydration of cement. Their results showed that oxidized CNCs greatly increased hydration heat releases and flexural strength. Hunek et al. [10] compared the effects of CNCs and CNFs on the various properties of concrete. The high aspect ratio of CNFs significantly increased concrete flexural strength, and the hydrophobic properties of the nanomaterials influenced frost resistance. As a result of hydration acceleration, the addition of CNFs increased the compactness of cement matrix and reduced the number and area of pores [11]. Cengiz et al. [12] used CNFs extracted from algae as an additive and found that the flexural strength of concrete increased by 270% compared with the blank group. The addition of CNFs can improve the mechanical properties of cement-based materials, but it should be noted that too high a CNF content will weaken concrete performance due to agglomeration of the nanomaterial. This was demonstrated in the research of Takasi et al. [13] who found that when the addition of CNFs increased from zero to 0.05%, the compressive strength increased, but when it changed from 0.05% to 0.2%, the compressive strength decreased. Goncalves et al. [14] found that CNFs significantly increased sulfate resistance; it also reduced the amount of ettringite used and decreased the expansion of the test piece.

As stated above, most research has concentrated on the short-term properties of CNF-reinforced cement-based materials, but studies on their durability are very limited. To the best of our knowledge, there is no literature investigating the effects of CNFs on the properties of pervious concrete. Specifically, CNFs show a rheological property-adjusting capacity and nano-reinforced effect for paste, which may have the potential to improve the bonding between aggregates for pervious concrete. In this study, we prepared samples of a new generation of high-performance pervious concrete by incorporating CNFs at 0%, 0.05%, 0.1%, 0.15% and 0.2% by weight of binder. The effects of CNFs on the mechanical properties, water permeability and frost resistance of pervious concrete were quantified. The rheological properties and heat of hydration of the samples were investigated to identify the matrix-strengthening mechanisms. We analyzed SEM images of the matrix and ITZ in specimens.

## 2. Experimental Program

### 2.1. Materials and Mixture Proportions

The binders were ordinary Portland cement (OPC, 42.5 grade), silica fume (SF), and class II fly ash (FA); their chemical composition and physical properties are shown in Table 1. The coarse aggregate (CA) was 5–10 mm limestone. A high-range water-reducing admixture (HRWRA) was used to adjust the workability of the pervious concrete.

**Figure 1:**
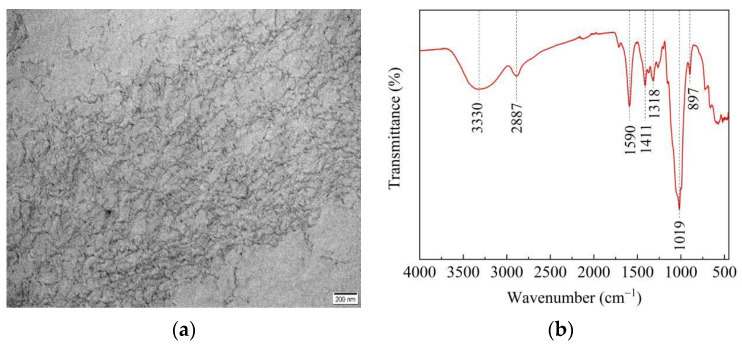
Characteristics of CNFs. (**a**) TEM image; (**b**) FTIR spectra.

The CNFs used in this study were provided by Shengquan Group Co., Ltd., Jinan, China, and the physical and mechanical properties of the CNF are tabulated in Table 2. Figure 1a is a transmission electron microscope (TEM) image of CNFs and shows that the nanofibers were 10–50 nm in diameter and 500–1000 nm long. Attenuated total reflection-Fourier transform infrared (ATR-FTIR) analysis was conducted using an iS50 FT-IR spectrometer (Thermo Fisher Scientific, USA), as shown in Figure 1b. The peak at 3330 cm^−1^ was related to the stretching vibrations in O–H from hydroxyl groups and the peak at 1590 cm^−1^ was characterized by the antisymmetric stretching vibrations of COO– from carboxylic salt [7,15]. CNFs were predispersed in water in order to disperse it uniformly into pervious concrete. The CNFs and water were mixed for 30 s with a shear stress at 30,000 rpm and then homogenized by magnetic stirring for 5 min. Homogeneous CNF suspensions (0.6 wt%) were obtained by ultrasonic dispersion.

Table 3 shows the mixture proportions; CNFs were added at proportions of 0.05%, 0.1%, 0.15% and 0.2% by weight of binder.

### 2.2. Test Methods

#### 2.2.1. Rheological Properties

**Figure 2:**
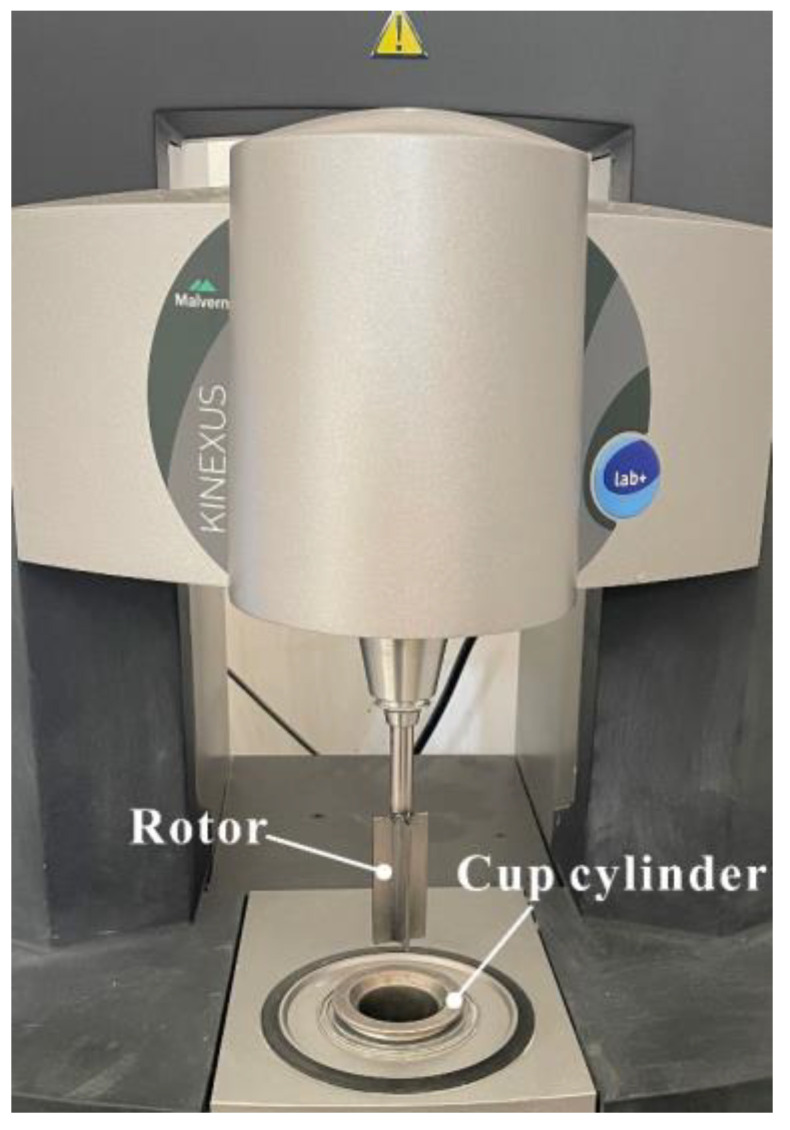
Rheological test setup.

The rheological properties of matrices (without aggregates) were assessed using a cylindrical rotational rheometer (Kinexus, Malvern, UK), and test setup is shown in Figure 2. The shear rate of the rotor increased from 0/s to 100/s and then decreased to 0/s. In total, 70 data points were recorded to define the shear stress–shear rate curve, and the modified Bingham model (Equation (1)) was used to characterize the rheological behavior of the matrix. Two rheological parameters, yield stress (*τ*_0_) and plastic viscosity (*μ*), were used to quantify the effect of CNF on the rheological properties of the matrix.

(1) $$\tau =\tau_{0}+\mu \gamma +c\gamma^{2}$$

where *τ* is shear stress (Pa), *γ* is shear rate (/s), *τ*_0_ is yield stress (Pa), *μ* is plastic viscosity (Pa·s), and *c* is the regression coefficient.

#### 2.2.2. Isothermal Calorimetry

**Figure 3:**
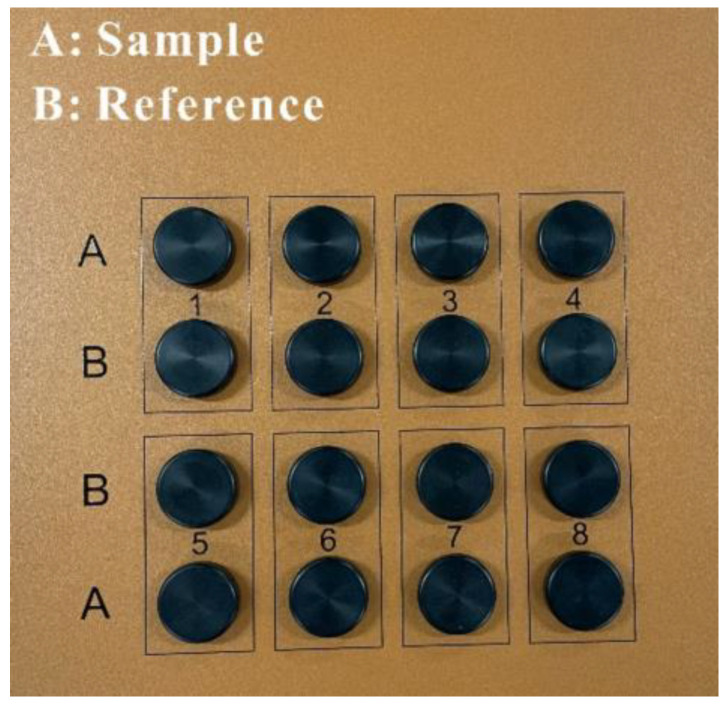
Test setup for isothermal calorimetry.

Isothermal calorimetry tests were conducted using a TAM air calorimeter (TA instruments, New Castle, DE, USA) to determine the effect of CNFs on the early hydration of matrices. The fresh paste sample (without aggregates) was sealed into an ampoule and placed into the calorimetric chamber at a temperature of 20 ± 0.1 °C. As shown in Figure 3, reference (water) was inserted into chamber A, and the sample was inserted into chamber B.

#### 2.2.3. Porosity and Water Permeability

Three specimen cubes with 100 mm sides were prepared for the porosity test. Porosity was calculated by Equation (2).

(2) $$P=(1-\frac{W_{2}-W_{1}}{\rho_{w}V_{s}})\times 100$$

where *P* is porosity (%), *W*_2_ is the weight of the specimens dried for 24 h (kg), *W*_1_ is the weight of the specimen submerged in water (kg) and measured using a hydrostatic balance, *ρ*_w_ is the density of water (kg/mm^3^), and *V_s_* is the volume of the specimen (mm^3^).

**Figure 4:**
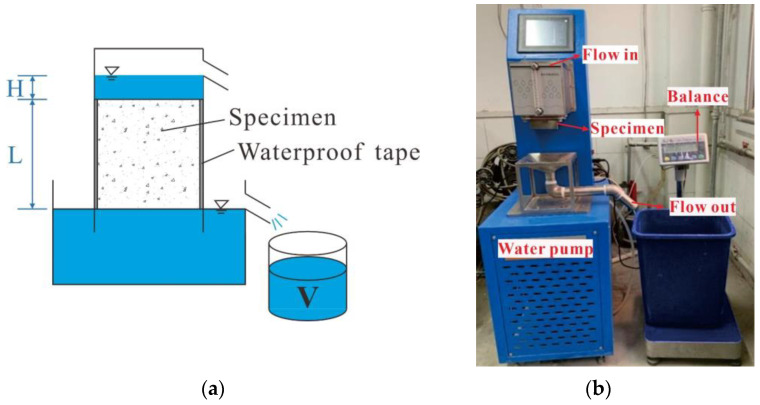
Test setup to determine the water permeability of pervious concrete. (**a**) Schematic diagram; (**b**) Device.

The water permeability of pervious concrete was quantified by a constant water head method [16]. The test setup is shown in Figure 4. The side surfaces of the 100 mm cube were wrapped with waterproof adhesive tape to ensure that water only permeated through the vertical cross-section. The permeability coefficient was determined using Equation (3).

(3) $$k=\frac{L}{H}\times \frac{V_{1}-V_{2}}{(t_{2}-t_{1})\times A}$$

where *k* is the permeability coefficient (mm/s), *V*_1_ is the initial liquid volume (mm^3^), *V*_2_ is the final liquid volume (mm^3^), *t*_2_ is the finishing time (s), *A* is the cross-sectional area of the specimen (mm^2^), *L* is the height of the specimen (mm), and *H* is the water head (mm).

#### 2.2.4. Mechanical Properties

Compressive strength and flexural strength were measured according to Chinese National Standard GB/T 50081-2019. The 28 d compressive strength was measured on 50 × 50 × 50 mm cubes; specimens were loaded at a rate of 0.5 MPa/s. Four-point flexural strength was measured using 100 × 100 × 400 mm prisms at 28 d at a loading rate of 0.5 MPa/s.

#### 2.2.5. Frost Resistance

**Figure 5:**
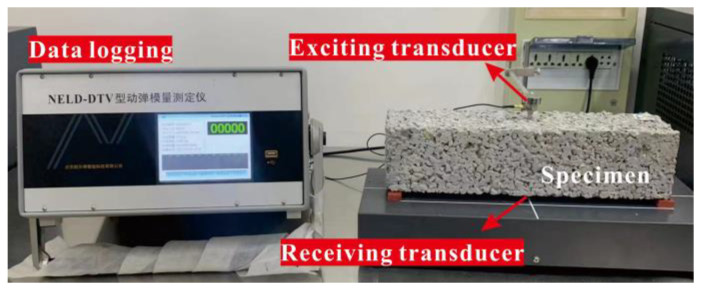
Test setup for dynamic elasticity modulus.

Frost resistance was determined by a rapid freeze and thaw (F–T) cycling test according to Chinese National Standard GB/T 50082-2009. Prisms with dimensions 100 × 100 × 400 mm were subjected to F–T cycling. In this test, the central temperature of a specimen was alternatively lowered from 5 ± 2 °C to −18 ± 2 °C and then raised from −18 ± 2 °C to 5 ± 2 °C. One cycle was completed within a 2–4 h period. Mass loss and dynamic elasticity modulus were calculated at intervals of 25 cycles. As shown in Figure 5, the dynamic elasticity modulus was measured using a resonance method. The relative dynamic elasticity modulus f was calculated by Equation (4).

(4) $$f=\frac{f_{i}^{2}}{f_{0}^{2}}\times 100$$

where *f*_0_ is the initial dynamic elasticity, and *f*_i_ is the dynamic elasticity after a multiple of 25 cycles.

#### 2.2.6. Scanning Electron Microscopy

The matrices and interfacial transition zones (ITZ) between matrices and aggregates were observed using scanning electron microscopy (SEM). The fresh fractured specimens were dried to a constant weight at 45 ºC and then sputtered with gold–palladium in a vacuum chamber for testing.

## 3. Results

### 3.1. Rheological Properties

Matrix rheology is critical in the paste coating of aggregates, and the thickness of a paste corresponds to its viscosity. A nonuniform coating thickness for aggregates leads to a weak ITZ and deterioration of mechanical properties.

**Figure 6:**
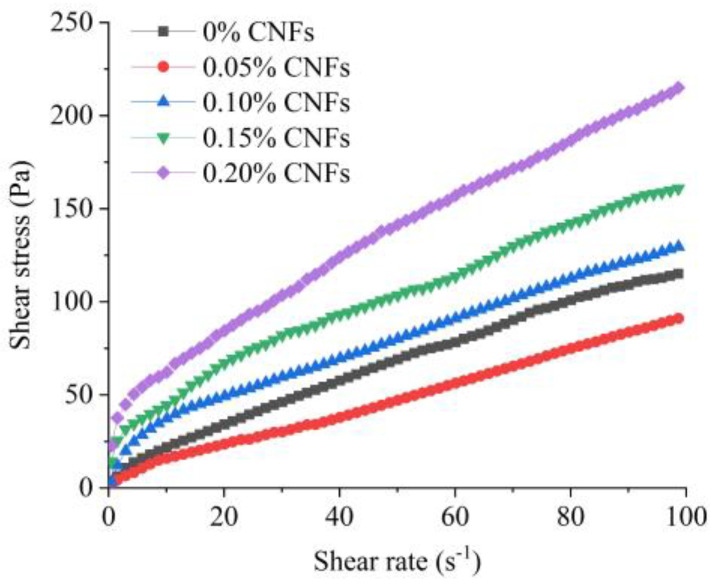
Shear stress–shear rate curves.

Figure 6 shows the effect of CNF addition on the shear stress–shear rate curves of matrices, and matrices showed a shear thinning behavior. Table 4 lists the values of the rheological parameters determined by the modified Bingham model. A correlation coefficient *R*^2^ > 0.99 indicates that the modified Bingham model fits the rheological behavior well. It was found that a 0.05% CNF addition slightly decreased yield stress and plastic viscosity of the matrix and that yield stress and plastic viscosity increased as the proportion of CNFs increased. The addition of the 0.1%, 0.15% and 0.2% CNFs increased yield stress from 6.85 Pa to 19.50, 28.37 and 38.04 Pa, respectively, corresponding to an increase of 184.7%, 314.2%, and 455.3%, respectively. Plastic viscosity increased by 0 to 69.8% when CNF addition increased from 0.1% to 0.2%. We observed that CNFs had a more significant effect on yield stress than on plastic viscosity.

The effect of CNFs on the rheological properties of the matrix is attributed to their hydrophilicity, which are similar to those of a viscosity-modifying agent (VMA). The hydroxyl and carboxylate groups on the surface of cellulose skeletons combine with water molecules to form hydrogen bonding, providing the capacity for water adsorption [6]. Moreover, CNFs may adsorb the surface of cement particles, thus increasing the friction between CNFs and cement inside fresh mixtures [8].

### 3.2. Isothermal Calorimetry

**Figure 7:**
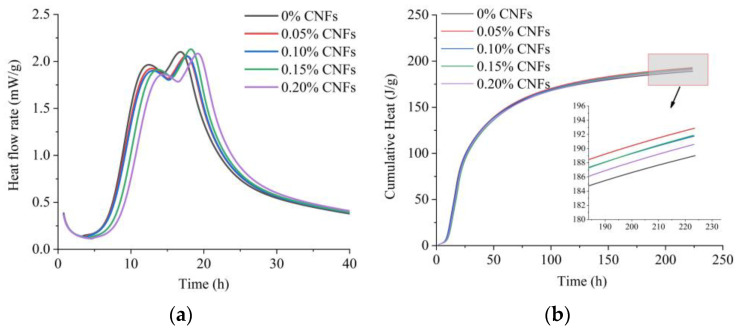
Isothermal calorimetric curves. (**a**) Heat flow; (**b**) Cumulative heat.

Figure 7 shows that the heat flow and cumulative heat released by matrices varied with CNF addition. Figure 7a shows that the effect of 0.15–0.2% CNF addition could be clearly observed within the first 10 h, and the delays in the first silicate hydration peak and secondary aluminate hydration peak were observed in all groups with added CNFs. Heat flow corresponding to the silicate hydration peak decreased due to the addition of CNFs. Although the aluminate hydration peak for 0.15% CNFs was higher, the aluminate hydration peaks for the 0.05%, 0.1% and 0.2% CNF addition were lower. Figure 7b shows the effect of CNF addition on cumulative heat release. CNFs increased the cumulative heat release within 200 h, and the 0.05% CNF addition resulted in the greatest value.

FTIR spectra showed that the surfaces of CNFs contained hydroxyl and carboxylate groups. The oxygen atoms in hydroxyl and carboxylate groups had unpaired electrons, and a hydrophilic complexation was formed when they reacted with calcium ions, which decreased the rate of formation of hydrated calcium silicate and calcium hydroxide [17]. The increase in cumulative heat can be attributed to the short-circuit diffusion (SCD) mechanism proposed by Cao et al. [18]. CNFs with adsorbed water can adsorb on the surface of cement particles and will release water and transport water to the unhydrated cement particles to promote hydration at later ages.

### 3.3. Water Permeability

**Figure 8:**
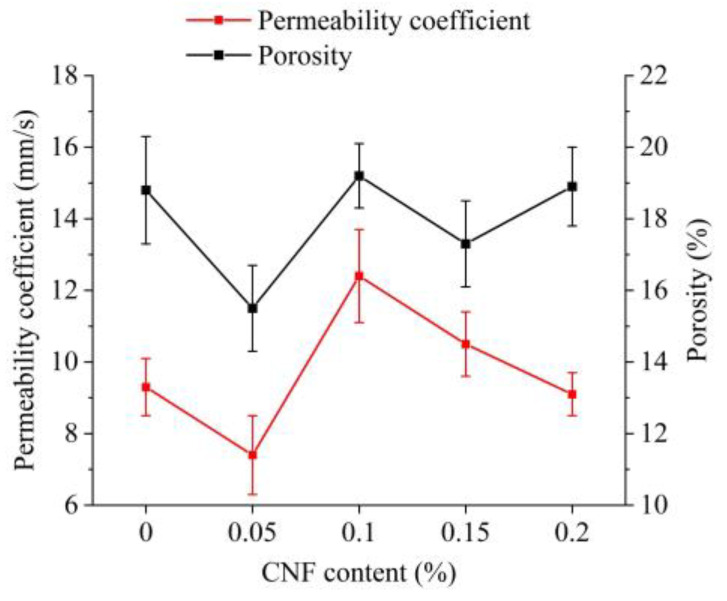
Porosity and permeability coefficient of pervious concrete.

The porosity of pervious concrete determines the water permeability coefficient that characterizes the concrete infiltration capacity. Total porosity and water permeability coefficients of different specimens are shown in Figure 8. The addition of 0.1% CNFs increased total porosity from 18.8% to 19.2% for a 2.1% increase and the permeability coefficient from 9.3 mm/s to 12.4 mm/s for a 33.3% increase. These were the greatest percentage increases of all groups. An addition of 0.05% CNFs resulted in 15.5% porosity and a permeability coefficient of 7.4 mm/s, which were the lowest values of all groups. As reported in Section 3.1, a 0.05% CNF addition resulted in the lowest values of plastic viscosity and yield stress. These values indicate insufficient surface tension of the slurry, which causes the slurry to settle and thus blocks the interconnected pores at the base of the specimen. For a 0.2% CNF addition, extremely high values of plastic viscosity and yield stress may lead to a situation similar to that described in Section 3.1, which is associated with a greater reduction in the permeability coefficient.

### 3.4. Mechanical Properties

**Figure 9:**
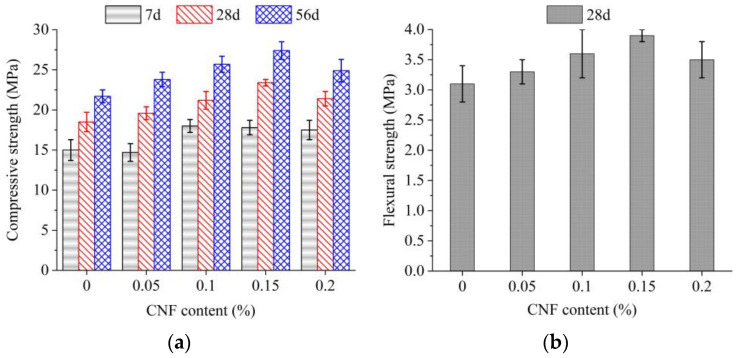
Development of compressive strength and flexural strength of pervious concrete. (**a**) Compressive strength; (**b**) Flexural strength.

The compressive strength and flexural strength of specimens in all groups are shown in Figure 9. Figure 9a shows the effect of CNF addition on the compressive strength at different curing ages. At 7 d, the groups with 0.1%, 0.15% and 0.2% CNF additions showed respective increases of 20%, 18.7% and 16.7% in compressive strength, and the group with the 0.05% CNF addition showed a slightly lower (−2%) compressive strength than did the baseline group. A similar trend in the effect of CNF addition on compressive strength was observed at 28 d and 56 d.

An addition of 0.05%, 0.1%, 0.15% and 0.2% CNFs resulted in respective increases in 28 d compressive strength of 5.9%, 14.6%, 26.5% and 15.7%. At 56 d, the addition of 0.05%, 0.1%, 0.15% and 0.2% CNFs resulted in respective increases in 56 d compressive strength of 23.8, 25.7, 27.4 and 24.9 MPa, corresponding to percentage increases of 9.7%, 18.4%, 26.6% and 14.7% compared to the 21.7 MPa compressive strength of the baseline group. Figure 9b shows the flexural strength at 28 d. An addition of 0.05%, 0.1%, 0.15% and 0.2% CNFs increased flexural strength from 3.1 MPa to 3.3 MPa, 3.6 MPa, 3.9 MPa and 3.5 MPa, respectively, corresponding to percentage increases of 6.5%, 16.1%, 25.8% and 12.9%. In all cases, the addition of CNFs increased both compressive strength and flexural strength after 28 d, with the greatest values given with a 0.15% CNF addition. When the CNF addition increased from 0.05% to 0.15%, the monotonically increasing strengths matched the higher heat release (Section 3.2). Moreover, the greatest strength might be linked to the buildup of CNF networks [6]. Due to the high aspect ratio and flexibility of CNFs, the threshold value of a 0.15% CNF addition could form the three-dimensional CNF networks. At a 0.15% CNF content, the rheological parameters also underwent an abrupt change. Consequently, CNF networks could bridge microcracks and hydrates, thus contributing to the highest strength for the 0.15% CNFs. CNFs also increased flexural strength by bridging microcracks in the matrix [19,20], resembling the bridging capacity in carbon nanofiber [21]. The decreased percentage increments in compressive and flexural strength for the 0.2% CNF addition may be related to inconsistent CNF dispersion into the matrix.

### 3.5. Frost Resistance

F–T damage is a critical concern in pervious concrete applications that are exposed to freezing, such as stadiums and sidewalks. Higher saturation makes pervious concrete frostbitten due to the network of many (semi-) connected pores, thus decreasing mass and reducing strength.

**Figure 10:**
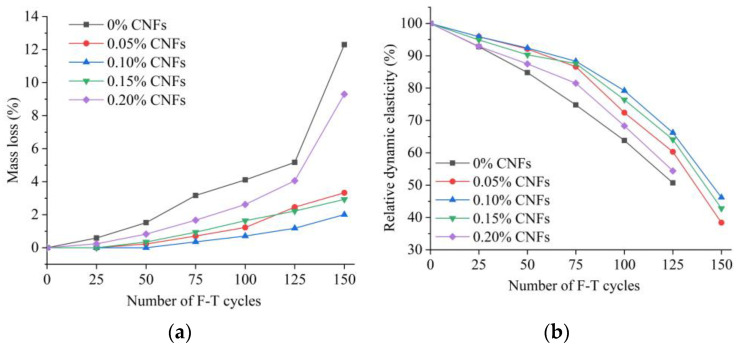
Mass loss and dynamic elastic modulus of pervious concrete subjected to 150 F–T cycles. (**a**) Mass loss; (**b**) Dynamic elastic modulus.

**Figure 11:**
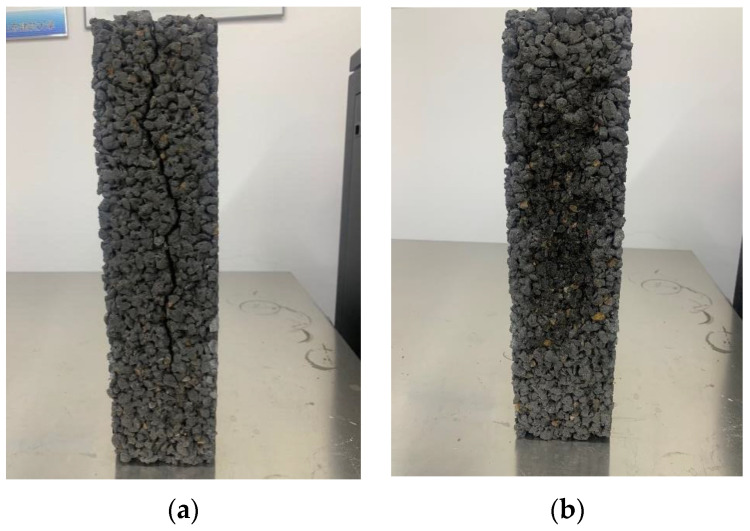
Cracks in specimens subjected to 150 F–T cycles. (**a**) 0.2% CNFs; (**b**) 0.1% CNFs.

Figure 10 shows the mass loss and change in the dynamic elastic modulus of pervious concrete containing CNFs for 150 F–T cycles. Figure 10a shows that the pervious concrete with added CNFs exhibited decreased mass loss and that resistance to the mass loss increased significantly in the groups with 0.05–0.15% added CNFs. In the case of the baseline group, the mass loss reached 12.3%, while for the 0.05%, 0.1% and 0.15% CNF additions, the mass loss was respectively 3.3%, 2.0% and 2.9%, corresponding to percentage decreases of 73.2%, 83.7% and 76.4%. Figure 10b shows that pervious concrete with a 0.05–0.15% CNF addition had a residual rate of approximately 40–50% after 150 F–T cycles, whereas the baseline and 0.2% CNF addition produced growing cracks (see Figure 11) after 125 F–T cycles, resulting in measurement failure for the dynamic elastic modulus in each case.

### 3.6. SEM Observations

**Figure 12:**
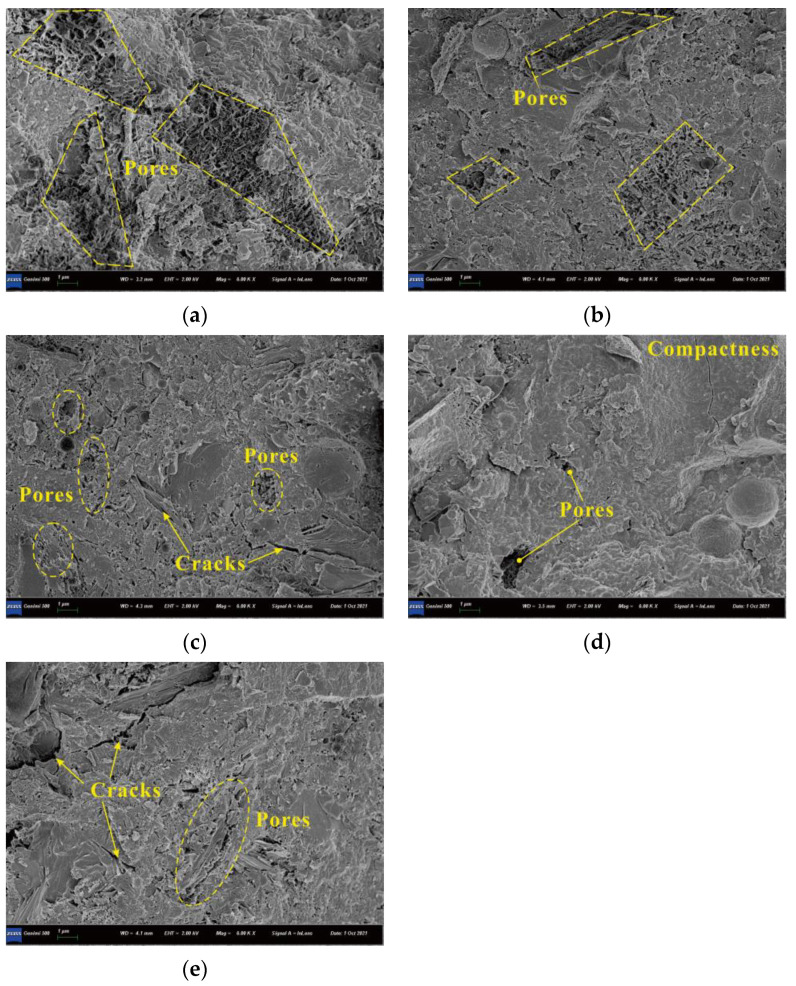
SEM images of matrices. (**a**) 0% CNF; (**b**) 0.05% CNFs; (**c**) 0.1% CNFs; (**d**) 0.15% CNFs; (**e**) 0.2% CNFs.

**Figure 13:**
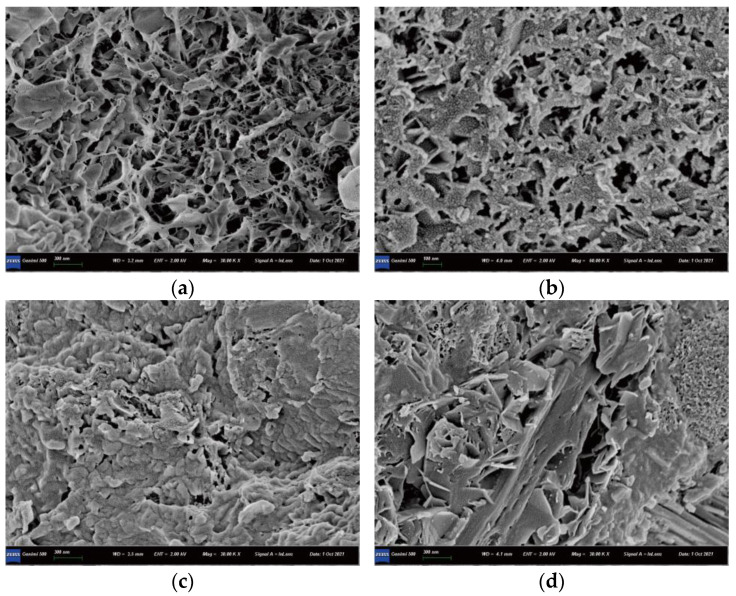
SEM images of the ITZ. (**a**) 0% CNF; (**b**) 0.05% CNFs; (**c**) 0.15% CNFs; (**d**) 0.2% CNFs.

The SEM images of matrices with various CNF additions are shown in Figure 12. Figure 12a–d show that the hydration products gradually became denser as CNF addition increased from 0.05% to 0.15%; the matrix structure without CNF showed some small pores, whereas that with 0.05–0.15% CNFs were more compact, which contributes to the improved strength. However, agglomerated CNFs might cause stress concentration in matrix, and thus minor cracks were observed with the 0.2% CNF addition (Figure 12e); it is agreed that cracks offer opportunities for water and chemical ion invasions, causing the poorest frost resistance among all CNFs mixtures. An ITZ of coarse cement matrices is considered to be the weakest region of the concrete. The microcracks in the ITZ gradually evolve into macrocracks, a process that decreases bearing capacity [22,23]. The hydration products of the ITZ are shown in Figure 13. The figure shows that the morphology of hydration products was transformed from a pore network structure to a compact structure as the CNF addition increased from 0% to 0.15%. The 0.15% CNF addition produced the densest hydration products, resulting in the strongest bonding between the aggregate and matrix; this was the best performing ITZ compared with the baseline, which is related to the highest strength and durability. The 0.1% and 0.15% CNF additions can therefore be considered optimal since they increase compressive strength and have a slighter decrease in durability compared to the no-CNF baseline. The 0.2% CNF addition can be regarded as an excessive quantity of CNFs that can cause flaws in the concrete [24].

## 4. Conclusions

Porosity, permeability, strength development and frost resistance of pervious concrete with additions of 0.05%, 0.1%, 0.15% and 0.2% CNFs were investigated. The major conclusions are summarized as follows.

1. The effects of the CNF addition on the porosity and permeability of pervious concrete were significant. The addition of 0.1% and 0.15% CNFs increased the permeability coefficient from 9.3 mm/s to 12.4 mm/s and from 9.3 mm/s to 10.5 mm/s, respectively, corresponding to increases of 33.3% and 12.9%. These increases were attributed to the increased mixture stability and homogeneity. This case could be explained by the corresponding optimal variation of plastic viscosity and yield stress of unadulterated matrices.
2. Although the addition of 0.05–0.2% CNFs significantly delayed the heat flow peak at 0–24 h, cumulative heat increased until 200 h. The cumulative heat for the 0.05–0.15% CNF additions was greater than that for the 0.2% CNF addition.
3. The addition of 0.05–0.2% CNFs increased compressive strength by 9.7–26.6% and flexural strength by 6.5–25.8%; the 0.15% CNF addition showed the maximum increase in both indicators.
4. When the CNF addition was 0.05%–0.15%, frost resistance of pervious concrete increased with the addition of CNFs, and mass loss was significantly reduced by 73.2%–83.7%; however, with the 0.2% CNF addition, care should be taken to avoid a significant mass loss close to the 0% CNF addition.
5. The SEM results showed that the CNF addition changed the morphology and porosity of the hydration products in the matrices and ITZs. The addition of the 0.05–0.15% CNFs increased hydration products and reduced the number of pores, resulting in denser matrices and stronger ITZs. When increased strengths and frost resistance were also considered, additions of 0.1% and 0.15% CNFs could be regarded as optimal.

The overall results prove the effectiveness of CNFs as a reinforced agent for improving the short-term strength and frost resistance of pervious concrete. In the future, experiments on long-term strength, sulfate attack and carbonization will be carried out to further study the effects of CNFs on the durability of pervious concrete. Moreover, the application of CNFs in more types of cement-based materials will be studied.

## Figures and Tables

**Table 1:** Chemical composition and physical and mechanical properties of binders.

Raw Materials	Chemical Composition (wt%)					OPC		
	CaO	SiO_2_	Al_2_O_3_	Fe_2_O_3_	MgO	Special Surface Area (m^2^/kg)	28 d Compressive Strength (MPa)	Flexural Strength (MPa)
OPC	63.6	21.2	3.2	3.0	1.0	353	47.8	7.9
FA	8.1	53.4	24.2	4.4	0.8			
SF	0.4	98.2	1.5	0.8	1.1			

**Table 2:** Physical and mechanical properties of CNF.

Properties	Length (mm)	Diameter (mm)	Density (g/cm^3^)	Tensile Strength (MPa)	Young’s Modulus (GPa)
CNF	10–50	500–1000	0.7	8000–10,000	100–200

**Table 3:** Mixture proportions (kg/m^3^).

Group ID	OPC	FA	SF	CA	HRWRA	Water	CNF
CNF-1	357	42	21	1470	0.42	117.6	0
CNF-2	357	42	21	1470	0.42	117.6	0.21
CNF-3	357	42	21	1470	0.42	117.6	0.42
CNF-4	357	42	21	1470	0.42	117.6	0.63
CNF-5	357	42	21	1470	0.42	117.6	0.84

**Table 4:** Rheological parameters of matrices calculated by the modified Bingham model.

CNF Content (%)	*τ*_0_ (Pa)	*μ* (Pa·s)	*c*	*R* ^2^
0	6.85	1.39	−0.003	0.999
0.05	5.99	0.80	−0.001	0.999
0.1	19.50	1.39	−0.003	0.999
0.15	28.37	1.76	−0.004	0.999
0.2	38.04	2.36	−0.006	0.998

## Data Availability

Not applicable.

## References

[1] Ćosić K., Korat L., Ducman V., Netinger I. (2015). Influence of aggregate type and size on properties of pervious concrete. Constr. Build. Mater..

[2] Agar-Ozbek A.S., Weerheijm J., Schlangen E., Van Breugel K. (2013). Investigating porous concrete with improved strength: Testing at different scales. Constr. Build. Mater..

[3] Li J., Zhang Y., Liu G., Peng X. (2017). Preparation and performance evaluation of an innovative pervious concrete pavement. Constr. Build. Mater..

[4] Tabatabaeian M., Khaloo A., Khaloo H. (2019). An innovative high performance pervious concrete with polyester and epoxy resins. Constr. Build. Mater..

[5] Montes F., Fu T., Youngblood J.P., Weiss J. (2020). Rheological Impact of Using Cellulose Nanocrystals (CNC) in Cement Pastes. Constr. Build. Mater..

[6] Hisseine O.A., Basic N., Omran A.F., Tagnit-Hamou A. (2018). Feasibility of Using Cellulose Filaments as a Viscosity Modifying Agent in Self-Consolidating Concrete. Cem. Concr. Compos..

[7] Liang L., Zhang X., Liu Q., Li X., Shang X. (2022). Cellulose Nanofibrils for the Performance Improvement of Ultra-High Ductility Cementitious Composites. Cellulose.

[8] Liang L., Yang J., Lv G., Lei Z., Li X., Liu Q. (2021). Surface-Functionalized Nanocelluloses as Viscosity-Modifying Agents in Engineered Cementitious Composites. Front. Mater..

[9] Cao Y., Zavaterri P., Youngblood J., Moon R., Weiss J. (2015). The Influence of Cellulose Nanocrystal Additions on the Performance of Cement Paste. Cem. Concr. Compos..

[10] Barnat-Hunek D., Szymańska-Chargot M., Jarosz-Hadam M., Łagód G. (2019). Effect of cellulose nanofibrils and nanocrystals on physical properties of concrete. Constr. Build. Mater..

[11] Bakkari M.E., Bindiganavile V., Goncalves J., Boluk Y. (2019). Preparation of Cellulose Nanofibers by TEMPO-Oxidation of Bleached Chemi-Thermomechanical Pulp for Cement Applications. Carbohydr. Polym..

[12] Cengiz A., Kaya M., Pekel Bayramgil N. (2017). Flexural stress enhancement of concrete by incorporation of algal cellulose nanofibers. Constr. Build. Mater..

[13] Takasi P. (2019). A Laboratory Investigation of Cement Based Materials with Cellulose Nanofibers. Doctoral Degree.

[14] Goncalves J., El-Bakkari M., Boluk Y., Bindiganavile V. (2019). Cellulose nanofibres (CNF) for sulphate resistance in cement based systems. Cem. Concr. Compos..

[15] Benselfelt T., Pettersson T., Wågberg L. (2017). Influence of surface charge density and morphology on the formation of polyelectrolyte multilayers on smooth charged cellulose surfaces. Langmuir.

[16] Xie X., Zhang T., Wang C., Yang Y., Bogush A., Khayrulina E., Huang Z., Wei J., Yu Q. (2020). Mixture proportion design of pervious concrete based on the relationships between fundamental properties and skeleton structures. Cem. Concr. Compos..

[17] Bentz D.P. (2006). Influence of water-to-cement ratio on hydration kinetics: Simple models based on spatial considerations. Cem. Concr. Res..

[18] Wu J., Guo L., Cao Y., Lyu B. (2022). Mechanical and fiber/matrix interfacial behavior of ultra-high-strength and high-ductility cementitious composites incorporating waste glass powder. Cem. Concr. Compos..

[19] Haque M.I., Ashraf W., Khan R.I., Shah S. (2022). A comparative investigation on the effects of nanocellulose from bacteria and plant-based sources for cementitious composites. Cem. Concr. Compos..

[20] Kamasamudram K.S., Ashraf W., Landis E.N. (2021). Cellulose nanofibrils with and without nanosilica for the performance enhancement of Portland cement systems. Constr. Build. Mater..

[21] Golewski G.L. (2018). Evaluation of morphology and size of cracks of the Interfacial Transition Zone (ITZ) in concrete containing fly ash (FA). J. Hazard. Mater..

[22] Lan Y., Zheng B., Shi T., Ma C., Liu Y., Zhao Z. (2022). Crack resistance property of carbon nanotubes-modified concrete. Mag. Concr. Res..

[23] Shi T., Liu Y., Zhao X., Wang J., Zhao Z., Corr D.J., Shah S.P. (2022). Study on mechanical properties of the interfacial transition zone in carbon nanofiber-reinforced cement mortar based on the PeakForce tapping mode of atomic force microscope. J. Build. Eng..

[24] Sun X., Wu Q., Lee S., Qing Y., Wu Y. (2016). Cellulose Nanofibers as a Modifier for Rheology, Curing and Mechanical Performance of Oil Well Cement. Sci. Rep..

